# Human Bocavirus Infections in Hospitalized Children and Adults

**DOI:** 10.3201/eid1402.070851

**Published:** 2008-02

**Authors:** Jean Longtin, Martine Bastien, Rodica Gilca, Eric Leblanc, Gaston de Serres, Michel G. Bergeron, Guy Boivin

**Affiliations:** * Centre Hospitalier Universitaire de Québec, Quebec City, Quebec, Canada; † Université Laval, Quebec City, Quebec, Canada; ‡ Institut National de Santé Publique du Québec, Quebec City, Quebec, Canada

**Keywords:** Bocavirus, Parvoviridae, diagnosis, respiratory infections, children, adults, research

## Abstract

Studies have reported human bocavirus (HBoV) in children with respiratory tract infections (RTIs), but only occasionally in adults. We searched for HBoV DNA in nasopharyngeal aspirates (NPAs) from adults with exacerbations of chronic bronchitis or pneumonia, from children hospitalized for acute RTIs, and from asymptomatic children during the winter of 2002–2003 in Canada. HBoV was detected in NPAs of 1 (0.8%) of 126 symptomatic adults, 31 (13.8%) of 225 symptomatic children, and 43 (43%) of 100 asymptomatic children undergoing elective surgery. Another virus was detected in 22 (71%) of the 31 HBoV-positive NPAs from symptomatic children. Two clades of HBoV were identified. The pathogenic role of HBoV in RTIs is uncertain because it was frequently detected in symptomatic and asymptomatic children and was commonly found with other viruses in symptomatic children.

## Abstract 2

The pathogenic role of this virus in infected children is unclear.

Human bocavirus (HBoV) is a newly described human virus closely related to bovine parvovirus and canine minute virus. It is currently classified in the genus *Bocavirus* within the family *Parvoviridae*. This virus was first identified in respiratory tract specimens from Swedish children with lower respiratory tract infections (RTIs) (*1*). Nucleic acid amplification has detected HBoV in respiratory samples of children with acute respiratory disease, with incidence rates ranging from 3% to 19% (*1*–*23*). However, the pathogenic role of HBoV is uncertain because other viruses have been frequently detected in HBoV-positive children with lower RTIs (range 37%–90%) (*2*,*3*,*7*,*9*–*11*,*20*–*22*). The objective of this study was to describe the incidence and clinical manifestations of HBoV infections in children and adults with respiratory tract symptoms, including a control group of children without symptoms.

## Materials and Methods

### Study Design

Respiratory samples from adults were obtained from a previous study conducted from December 2002 to April 2003 at 3 university-affiliated hospitals in the province of Quebec, Canada (*24*). Two groups of patients were enrolled: those >40 years of age with chronic obstructive pulmonary disease (COPD) who came to emergency departments with exacerbation of their illness (including patients with pneumonia), and those >18 years of age without COPD who were admitted to the hospital with a diagnosis of community-acquired pneumonia. Patients were excluded from the study if they came to the hospital >7 days after onset of symptoms.

Respiratory samples from children were obtained from a case-control study, the results of which have been published (*25*). Participants included children <3 years of age who were hospitalized from December 2002 to April 2003, at Laval University Hospital Center in Quebec City, Quebec, Canada. Case-patients were children admitted for an acute RTI (mostly bronchiolitis, pneumonitis, and laryngotracheobronchitis) who had a nasopharyngeal aspirate (NPA) collected as part of investigation of their illness. A specific questionnaire was completed at admission by a research nurse in the presence of the parents. At the end of hospitalization, charts of the children were reviewed to collect clinical and laboratory data. Eligible controls were children hospitalized during the same period for any elective surgery (ear, nose, and throat surgeries in 71% of the cases). These children had no concomitant respiratory symptoms or fever at admission. The study nurse obtained a signed consent from parents and an NPA was obtained during surgery. The original studies were reviewed and approved by the ethics committees of all participating healthcare centers.

### Laboratory Testing

All pediatric (from case-patients and controls) and adult (case-patients only) NPA specimens were previously analyzed by using a multiplex real-time PCR assay for influenza A and B viruses, human respiratory syncytial virus (hRSV), and human metapneumovirus (hMPV) (*24*,*25*). For symptomatic children, viral cultures and antigen detection assays were performed upon request by the treating physician. Remaining specimens were frozen at –80°C until subsequent HBoV PCR studies.

Nucleic acids were extracted from 200 μL of NPA by using the QIAamp viral RNA Mini Kit (QIAGEN, Inc., Mississauga, Ontario, Canada). A duplex HBoV PCR (TaqMan assay) was used to amplify conserved regions of NP-1 and NS-1 genes as described (*14*), except that the NS-1 forward primer was replaced with primer 5′-TAG TTG TTT GGT GGG ARG A-3′. Probes were labeled with 6-carboxyfluorescein (FAM) or tetrachloro-6-carboxyfluorescein (TET) at the 5′ end and with a quencher at the 3′ end. Amplicons were 81 bp (NP-1) and 74 bp (NS-1), respectively. Duplex amplification was conducted by using 1 µmol/L NS-1 forward primer and 0.4 µmol/L NS-1 reverse primer and the 2 NP-1 primers. Taqman probes were used at concentrations of 0.1 mmol/L for the NP-1 gene and 0.2 mmol/L for the NS-1 gene (*14*). The amplification master mixture consisted of 2.5 mmol/L MgCl_2_, 3.33 mg/mL bovine serum albumin, 0.2 mmol/L of each of the 4 deoxynucleotide triphosphates (Amersham Biosciences, Uppsala, Sweden), 10 mmol/L Tris-HCl, 50 mmol/L KCl, 0.625 U Promega Taq DNA polymerase (Fisher Scientific, Markham, Ontario, Canada) combined with TaqStart antibody (BD Biosciences Clontech, Palo Alto, CA, USA), and 3 μL DNA in a final volume of 25 μL. PCR amplification (180 s at 94°C and 45 cycles for 10 s at 95°C, 30 s at 58°C, and 30 s at 72°C) was performed in a Smart Cycler thermal cycler (Cepheid, Sunnyvale, CA, USA). A PCR extension step of 5 min at 72°C was performed at the end of the cycling protocol. An HBoV infection was defined by a positive PCR result for NP-1 and NS-1. The duplex assay had a sensitivity of 10 genome copies for NP-1 and NS-1 gene targets on the basis of quantification analysis of positive control plasmids.

Half of the HBoV-positive samples were randomly selected for phylogenetic analysis, which consisted of amplifying and sequencing a 842-bp region of the VP1/VP2 genes as described (*6*). The VP1/VP2 nucleotide sequences from this study, as well as prototype sequence type (ST)1 and ST2 (*1*), were entered into a multiple alignment generated by ClustalW software version 1.83 (www.molecularevolution.org/software/clustalw) and corrected through final visual inspection with the SeqLab application (Wisconsin package version 10.3; Accelrys, San Diego, CA, USA). Phylogenetic analyses were conducted with the MEGA version 3.1 software (*26*) by using the distance method and the neighbor-joining algorithm with Kimura-2 parameters. Topologic accuracy of the tree was evaluated by using 1,000 bootstrap replicates.

### Statistical Analysis

Proportions of clinical characteristics in different groups of patients were compared by using the χ^2^ test or the Fisher exact test. The Wilcoxon nonparametric test was used to compare age distribution and length of stay. Analyses were performed by using SAS software version 9.1 (SAS Institute, Inc., Cary, NC, USA).

## Results

HBoV DNA was detected in NPA samples from 1 (0.8%) of 126 symptomatic adults (71 years of age) and from 31 (13.8%) of 225 symptomatic children (mean age 17 months, median age 15 months). However, HBoV was detected more frequently (43%, p<0.001) in the 100 asymptomatic control children (mean age 22 months, median age 23 months). Another virus was detected in 22 (71%) of 31 HBoV-positive NPAs from symptomatic children. The virus most commonly co-isolated with HBoV was hRSV (16/31, 52%), followed by influenza A/B (3 cases), hMPV (3 cases), adenovirus (1 case), and parainfluenza virus (1 case). Two children were infected with 2 other viruses in addition to HBoV. The median age of symptomatic children with HBoV infection (15 months) was significantly greater than that of symptomatic children without HBoV infection (8 months; p<0.0001). The hospital length of stay was similar for children positive for HBoV DNA (mean 5.1 days, median 4 days) and those negative for HBoV DNA (mean 6.6 days, median 3 days) (p = 0.9).

Clinical characteristics of HBoV-positive children are summarized in the Table. There were significantly fewer bronchiolitis episodes in children infected only with HBoV than in children infected only with hRSV (p<0.0001). None of the children with single HBoV infections and only 2 (6%) of all 31 HBoV-infected children were admitted to the intensive care unit. In the control group of asymptomatic children who underwent elective surgery, ear, nose, and throat surgery was more frequently performed in children with HBoV infections (36/43, 84%) than in children without HBoV infections (35/57, 61%) (p = 0.014). Ear, nose, and throat elective surgeries consisted mainly of myringotomies, adenoidectomies, and tonsillectomies.

**Table:** Clinical characteristics of symptomatic children with respiratory tract infections*

Characteristic	HBoV, all infections (n = 31)	HBoV, single infections (n = 9)	hRSV, single infections (n = 97)	hMPV, single infections (n = 12)	Influenza A/B virus, single infections (n = 3)
Pneumonia, no. (%)	13 (42)	4 (44)	17 (18)	1 (8)	1 (33)
Bronchiolitis, no. (%)†	13 (42)	1 (11)	83 (86)	5 (42)	2 (67)
Laryngotracheobronchitis, no. (%)	0	0	0	0	0
Otitis media, no. (%)	19 (61)	4 (44)	58 (60)	7 (58)	1 (33)
Median (mean) length of stay, d	3 (6.6)	3 (3.6)	4 (4.6)	3 (6.7)	4 (4.0)
Admission to ICU, no. (%)	2 (6)	0	10 (10)	0	0
Underlying cardiopulmonary disorders, no. (%)‡	3 (10)	0	11 (11)	3 (25)	3 (100)
Prematurity, no. (%)	2 (6)	0	10 (10)	0	0

*HBoV, human bocavirus; hRSV, human respiratory syncytial virus; hMPV, human metapneumovirus; ICU, intensive care unit. †p<0.0001 for comparison of single infections. ‡p = 0.0014 for comparison of single infections.

The 1 adult with an HBoV infection was a 71-year-old man (a smoker) who came to the hospital for a COPD exacerbation and was treated with systemic corticosteroids and antimicrobial drugs. No other microbiologic agents (bacteria or viruses) could be identified in his sputum or NPA. He was hospitalized for 11 days.

Sequence analysis of the HBoV VP1/VP2 genes performed on ≈50% of HBoV-positive specimens showed 2 distinct clades of viruses (Figure). These genotypes clustered with the original strains described by Allander et al. (ST1, GenBank accession no. DQ000495, and ST2, GenBank accession no. DQ000496) (*1*). There was no temporal link between the clades because both were equally distributed throughout the study period. No obvious relationship was found between clades and the presence or absence of symptoms.

**Figure:**
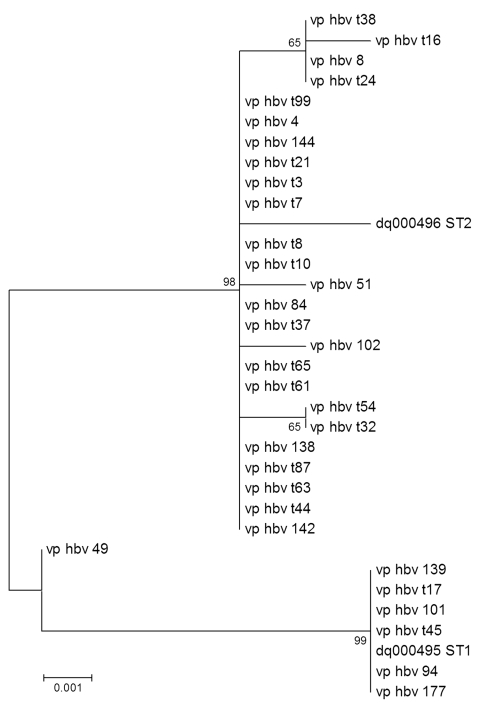
Phylogenetic tree of human pediatric bocavirus strains from Quebec City, Quebec, Canada. Patient numbers beginning with the letter t indicate asymptomatic (control) children. Strains from Sweden (sequence type [ST] 1, GenBank accession no. DQ000495, and ST2, GenBank accession no. DQ000496) are included (*1*). Numbers along branches are bootstrap values from 1,000 replicates. Scale bar shows 1 substitution for every 1,000 nucleic acid residues.

## Discussion

Results from our study indicate that HBoV was rarely detected in adults with respiratory symptoms but was frequently detected in symptomatic and asymptomatic children during the 2002–2003 winter season. HBoV was detected in NPA samples from 1 (0.8%) of 126 symptomatic adults, 31 (13.8%) of 225 symptomatic children, and 43 (43%) of 100 asymptomatic children. Another virus was detected in 22 (71%) of 31 HBoV-positive samples from symptomatic children. Overall, these data do not support a pathogenic role for HBoV in acute RTIs in children.

The full spectrum of clinical diseases associated with HBoV infections and the epidemiology of this new parvovirus are not fully understood. This is particularly true for adult patients in whom few studies have been performed. Allander et al. (*1*) found no HBoV DNA in 112 culture-negative NPA samples from adults with respiratory symptoms. Bastien et al. (*5*) reported an overall rate of infection of 1.5% in respiratory samples negative for other viruses, with no differences between age groups. Maggi et al. (*16*) reported only 1 HBoV-positive sample from an adult with lymphoma in 62 bronchoalveolar lavages (BALs). These investigators also tested 22 nasal swabs from adults with persistent asthma symptoms and found no samples positive for HBoV. Fry et al. (*10*) identified HBoV DNA in 1% of adults >20 years of age hospitalized with pneumonia in Thailand. Kupfer et al. (*27*) described a case of HBoV infection associated with severe atypical pneumonia in a patient with non-Hodgkin lymphoma who was also infected with cytomegalovirus in a BAL sample. We found 1 case of HBoV infection in an adult, which represented 0.8% of the tested population. The HBoV-positive adult did not show immunosuppression but was treated with corticosteroids for a COPD exacerbation. Overall, our results are consistent with those of previously described studies and support the fact that HBoV infection is rare in adults but may occur more frequently in those with other illnesses or immunosuppression.

Studies have reported HBoV DNA in 3%–19% of children with RTIs. Rates of detection tend to be higher in children <1 year of age (*4*,*10*). The incidence of HBoV infections also tends to be higher in samples from the lower respiratory tract, such as NPA or BAL (4.4%–19%) (*2*,*7*,*9*,*11*,*13*,*19*,*21*,*22*), than in nasal swabs (1%–6%) (*10*,*15*,*16*,*18*). The percentage of co-pathogens in our HBoV-positive children (71%) was comparable with those reported in the literature, with rates of co-infections ranging from 35% to 90% (*2*,*3*,*7*,*9*–*11*,*20*–*22*). Moreover, co-infecting viruses detected in conjunction with HBoV in our population were similar to those described in other studies, i.e., hRSV, influenza A virus, and adenovirus (*11*,*23*).

The high frequency of HBoV detection (43%) in our asymptomatic children contrasts with the results of the few other studies that included a control group of asymptomatic children. Fry et al. (*10*) detected HBoV DNA in only 1% of nasal swabs from asymptomatic patients. Maggi et al. (*16*) did not detect HBoV DNA in nasal swabs from 51 asymptomatic children (including 30 healthy infants with a mean age of 6 months and 21 preadolescent healthy children with a mean age of 12.8 years). However, these studies analyzed nasal swabs instead of NPA or BAL samples for HBoV detection, which may result in lower rates of viral detection, as shown in symptomatic persons. Allander et al. (*2*) did not detect HBoV in any of 64 asymptomatic children (median age 4.1 years, range 5 months to 14 years) but used nasal swabs in asymptomatic patients and NPA samples in symptomatic patients. Furthermore, their control group was also older than our population (mean 18.6 months, median 18 months). Kesebir et al. (*12*) did not detect any HBoV DNA in nasal washes from 96 asymptomatic children <2 years of age seen at a clinic compared with 22 (5.2%) of 425 various samples from symptomatic children sent to a hospital clinical laboratory. None of the previous studies used a control group consisting of children matched for age and week of admission and analyzed the same type of respiratory samples for cases and controls.

Our positive results for HBoV were confirmed by using 2 sets of PCR primers targeting different genes (NP1 and NS1) in a duplex PCR assay and by subsequent testing with a third set of primers (VP1/VP2) for sequencing. Also, sample preparation and PCR amplification were performed in separate laboratory areas following the stringent quality control program of our institution. Thus, it is unlikely that our positive results were due to PCR cross-contamination. Our method was also very sensitive (detection limit = 10 genome copies), which probably enabled an increased infection rate compared with previous reports. We cannot exclude the possibility that prior RTIs (in the few weeks preceding sampling) occurred in our asymptomatic children hospitalized for an elective surgery or that HBoV could establish a prolonged infection in children compared with other respiratory viruses. However, the 3× higher detection rate in controls than in symptomatic children make these explanations unlikely. We did not quantify HBoV DNA load in samples from our study, which could have been different between asymptomatic and symptomatic children. Nevertheless, we detected hRSV, hMPV, and influenza virus RNA in <1% of the same NPA samples from those asymptomatic children compared with a rate of 43% for HBoV DNA (*25*). At the very least, our results should raise concerns about the pathogenic role of HBoV in children.

We detected 2 HBoV genotypes circulating at the same time in both symptomatic and asymptomatic children during the winter of 2002–2003 in Quebec. This result is consistent with findings of other groups from North America and Europe during 2002–2004 and highlights the fact that HBoV lineages do not appear to be geographically clustered (*1*,*6*,*9*,*12*). The seasonality of HBoV infection is still a matter of debate, but it seems to involve primarily the colder months of the year (*9*,*20*,*21*). However, most studies, including ours, were performed during the typical respiratory virus season, which may have introduced a bias. Additional studies are needed to address the prevalence of HBoV outside the respiratory virus season and its role in nonrespiratory syndromes. Moreover, the possibility that this virus might be transmitted and isolated in the respiratory tract, but could cause viremia and other clinical syndromes such as gastroenteritis, should be investigated. Vicente et al. analyzed 527 stool samples from children with gastroenteritis and no respiratory symptoms and found a positivity rate of 9.1% for HBoV (with a co-infection rate of 58%) (*22*).

In conclusion, our study shows that HBoV was frequently detected in both symptomatic and asymptomatic children during the winter of 2002–2003 in Quebec City. Conversely, this virus was rarely found in the adult population during the same period. Further studies are needed to establish whether this recently described parvovirus is pathogenic by using well-matched control groups and sequential samples to detect viral persistence.
